# Supplementary material for: Human in vitro-induced IL-17A+ CD8+ T-cells exert pro-inflammatory effects on synovial fibroblasts
Source: Clin Exp Immunol. 2023 Jun 27;214(1):103–19. doi: 10.1093/cei/uxad068 (PMC10711358; doi:10.1093/cei/uxad068)
Supplement: uxad068_suppl_Supplementary_Figures [file uxad068_suppl_supplementary_figures.pdf]

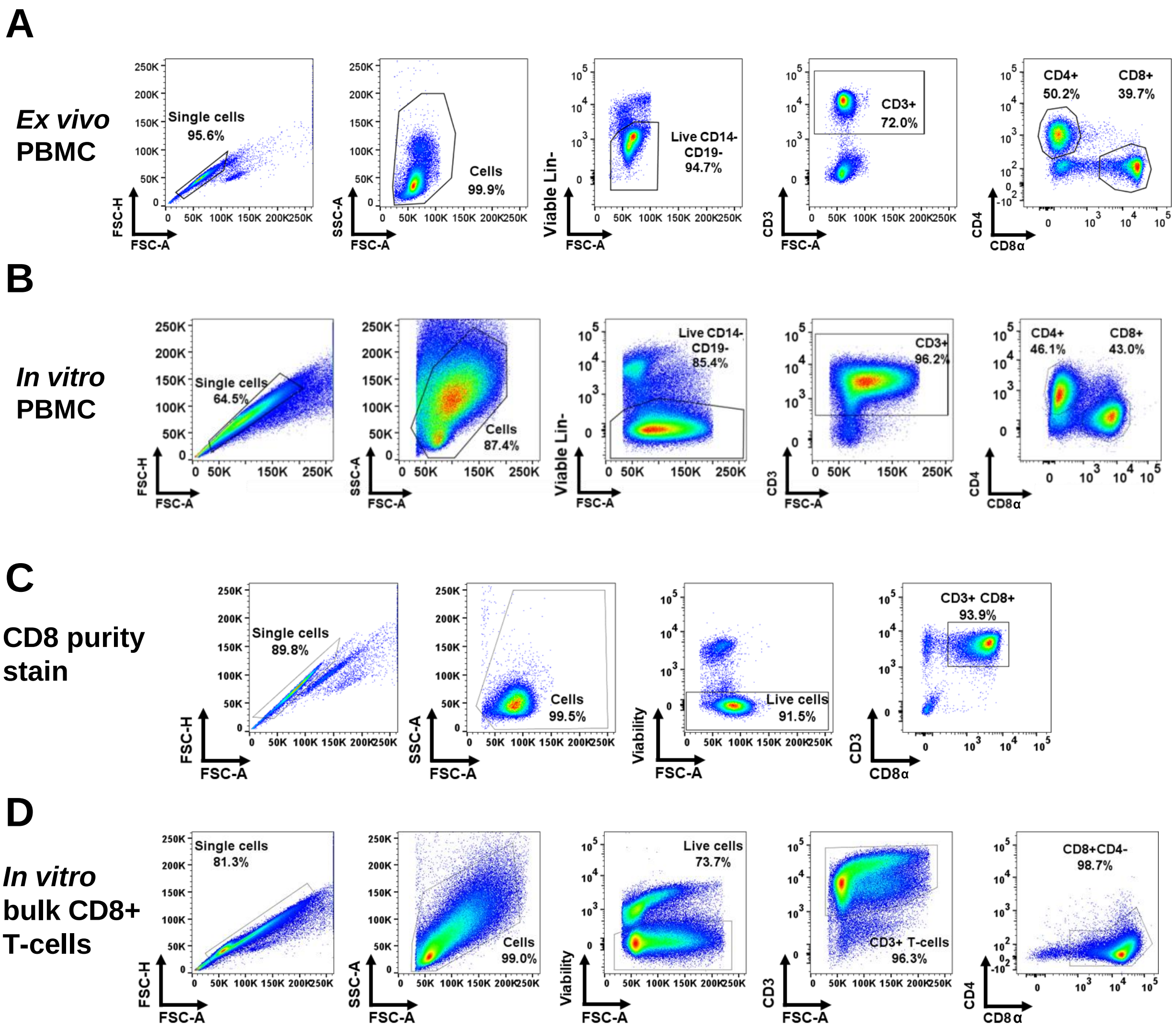

Supplementary Figure S1. Gating strategies for CD8+ and CD4+ T-cell identification within PBMC *ex vivo* or following *in vitro* cell culture. Healthy donor whole PBMC, either *ex vivo* **(A)** or following *in vitro* culture **(B)** were first gated on single cells and FSC/SSC. Dead cells and CD14+ cells were then excluded, followed by gating on CD3+ T-cells, from which CD8+ or CD4+ T-cell subsets were identified. **(C)** Representative purity staining for CD8+ T-cells isolated by magnetic bead separation from whole PBMC. Average CD8+ T-cell purity was 93% as determined by flow cytometry. **(D)** Gating strategy applied to identify live CD3+ CD8+ T-cells in CD8+ T-cell cultures following 3 day *in vitro* culture.

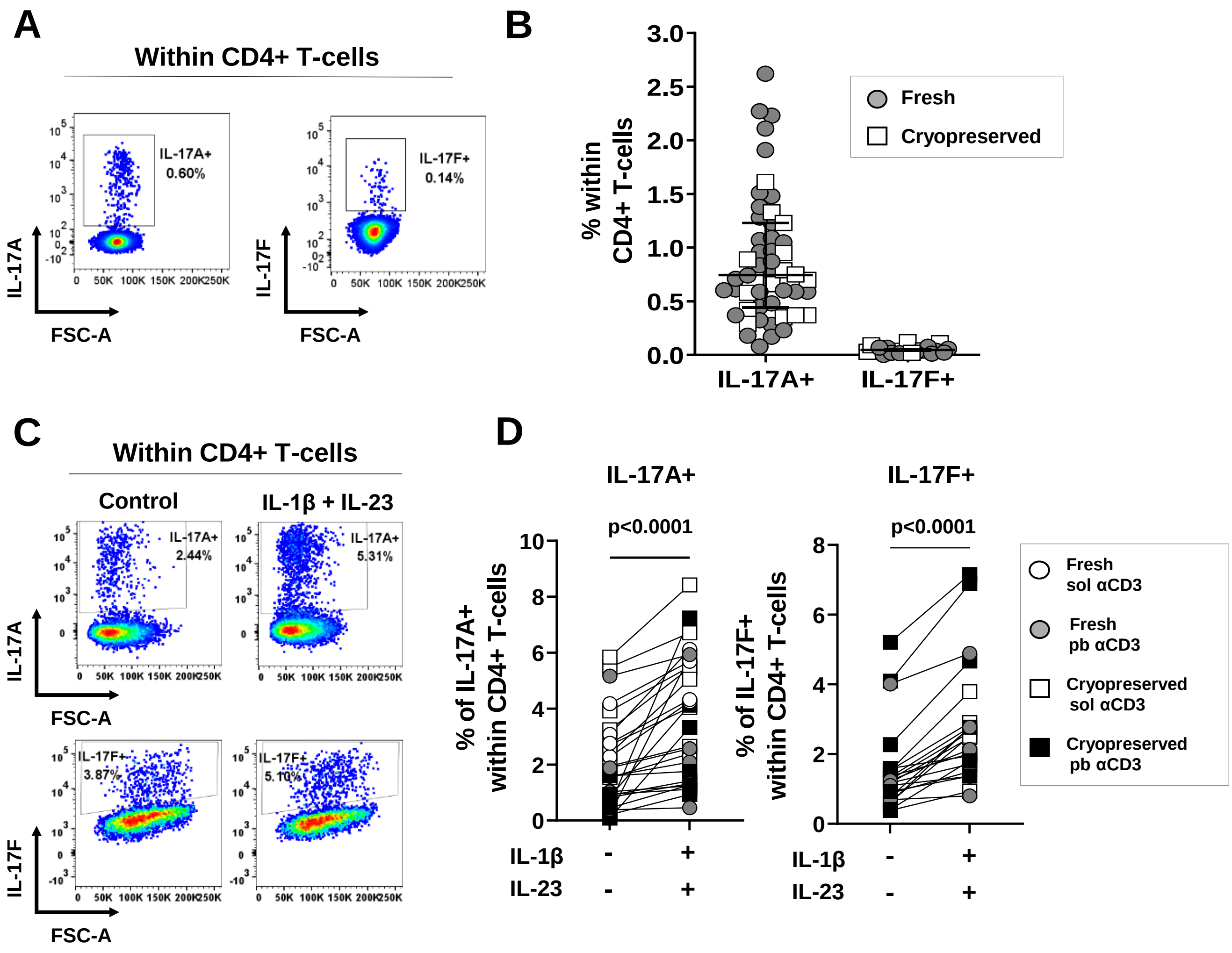

Supplementary Figure S2. Type-17 polarising conditions promote IL-17A and IL-17F expressing CD4+ T-cells. (A, B) Freshly isolated (circles) or cryopreserved (squares) healthy donor PBMC were stimulated *ex vivo* for 3 hours with PMA/ionomycin in the presence of GolgiStop for assessment of intracellular IL-17A and IL-17F cytokine expression by CD4+ T-cells using flow cytometry. Representative staining plots **(A)** and cumulative data **(B)** show frequencies of IL-17A+ and IL-17F+ cells within live CD3+ CD4+ T-cells from independent donors (n=50 and n=22, respectively). Data plotted as median ± IQR. **(C, D)** Fresh (circles) or cryopreserved (squares) healthy donor PBMC were cultured for 3 days with either plate-bound (filled symbols) or soluble (open symbols) anti-CD3 mAb and soluble anti-CD28 mAb in the absence (control) or presence of hrIL-1β and hrIL-23. After 3 days cells were re-stimulated with PMA/ionomycin in the presence of GolgiStop for detection of intracellular cytokine expression by CD4+ T-cells. Representative staining plots **(C)** and cumulative data **(D)** show frequencies of IL-17A+ and IL-17F+ cells within live CD3+CD4+ T-cells from independent donors (n=27 and n=22, respectively). Statistical analysis performed using Wilcoxon matched-pairs signed rank test.

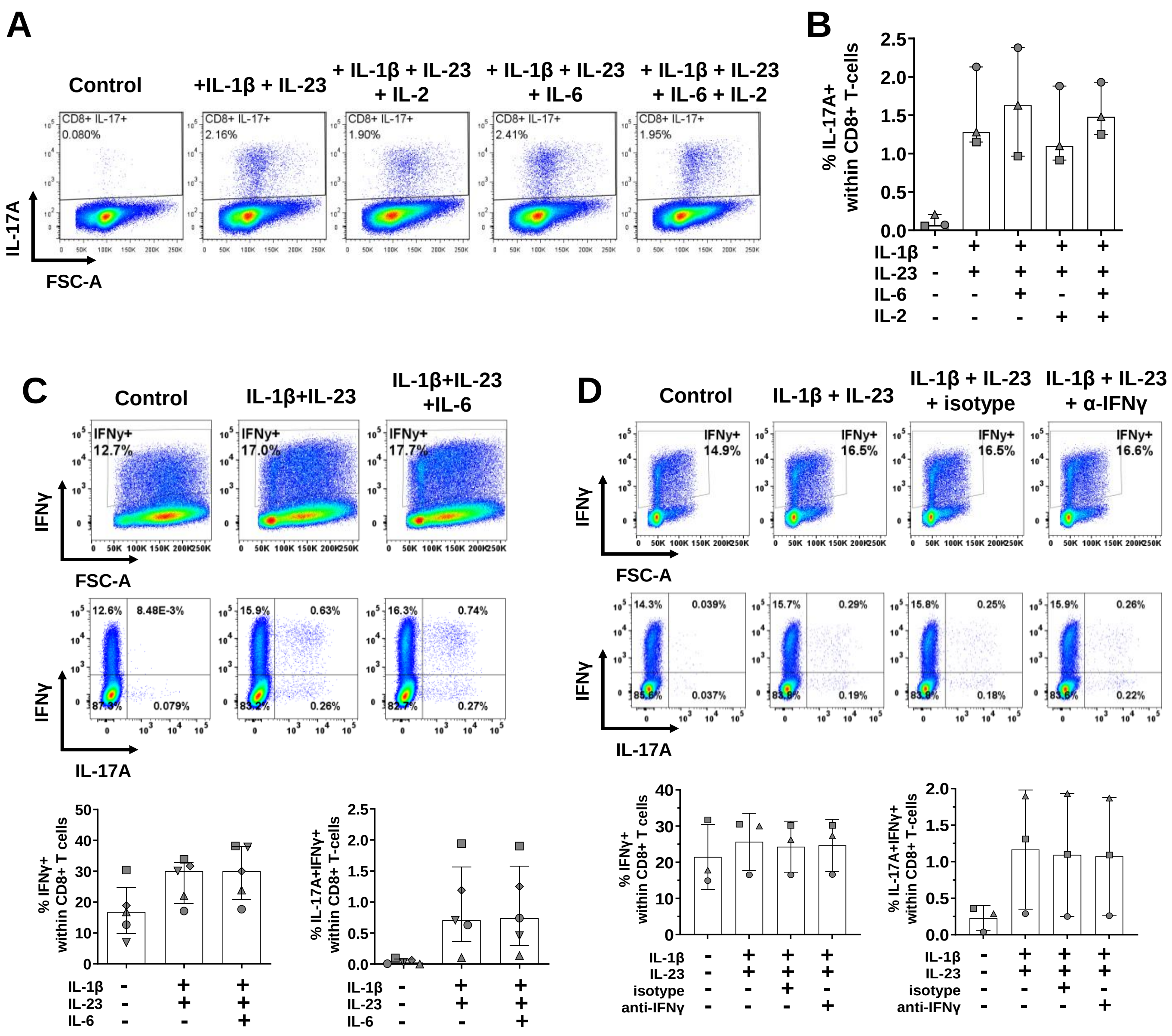

Supplementary Figure S3. Addition of hrIL-6 and/or hrIL-2 to *in vitro* cultures in combination with IL-1β and IL-23 does not further increase the frequency of induced IL-17A+ CD8+ T-cells. Healthy donor purified CD8+ T-cells were cultured in the presence of either anti-CD3/CD28 beads (1:10 bead to cell ratio) **(A, B)** or plate-bound anti-CD3 and soluble anti-CD28 mAbs **(C, D)** alone (control) or in the presence of hrIL-1β and IL-23 with or without rhIL-6 (20ng/ml) **(A-C)** or in the absence or presence of anti-IFNγ or isotype control mAb (5 µg/ml) **(D)** for 3 days. **(A, B)** On day 3, media containing recombinant cytokines was replenished and supplemented with or without hrIL-2 and cells cultured for an additional 3 days. On day 6 **(A, B)** or day 3 **(C, D)**, cells were re-stimulated for 3 hours with PMA/ionomycin in the presence of GolgiStop and intracellular cytokine expression assessed by flow cytometry. Representative staining plots and cumulative data showing the frequencies of IL-17A+ CD8+ T-cells **(A, B)** or single IFNγ+ and IFNγ+IL-17A+ CD8+ T-cells **(C, D)** across culture conditions. Data plotted as median ± IQR (n=3, each symbol corresponds to an independent donor).

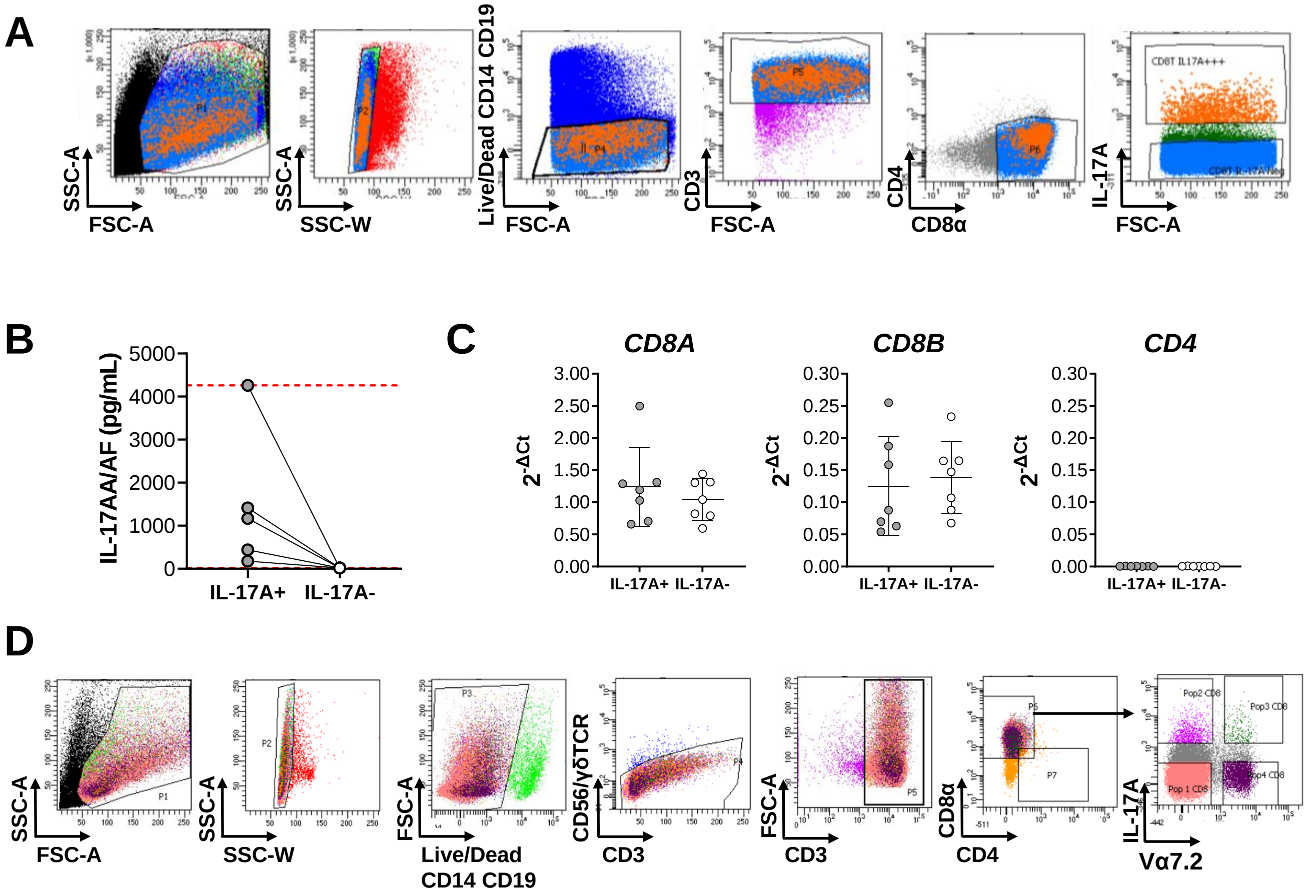

Supplementary Figure S4. Sorting of IL-17A-secreting CD8+ T-cells using an IL-17A cytokine secretion assay. Bulk CD8+ T-cells were cultured under type-17 polarising conditions for 3 days followed by 1.5 hours re-stimulation with PMA/ionomycin to allow for IL-17A secretion and capture using an IL-17A cytokine secretion assay. IL-17A secreting (IL-17A+) and non-secreting (IL-17A-) CD8+ T-cell subsets were FACS sorted according to surface marker and IL-17A detection antibody staining. Representative gating strategies used to identify **(A)** IL-17A+ and IL-17A- CD8+ T-cells. **(B)** Sorted IL-17A+ and IL-17A- T-cells were cultured for 20 hours in culture medium to generate supernatants which were analysed for IL-17AA/AF by Luminex assay (n=5). **(C)** Dot plots show absolute (normalised) mRNA expression levels of *CD8A, CD8B* and *CD4* within the indicated CD8+ T-cell populations as quantified by qPCR array (n=7 independent donors). Expression was normalised to housekeeping genes *B2M* and *PPIA*. Gene expression is reported as mean ± SD. **(D)** Representative gating strategy used to identify IL-17A+ Vα7.2- and IL-17A+ Vα7.2+ populations by CSA-FACS sorting as detailed above. Sorted IL-17A+ subsets were cultured for 20 hours in culture medium to generate supernatants for Luminex assay.

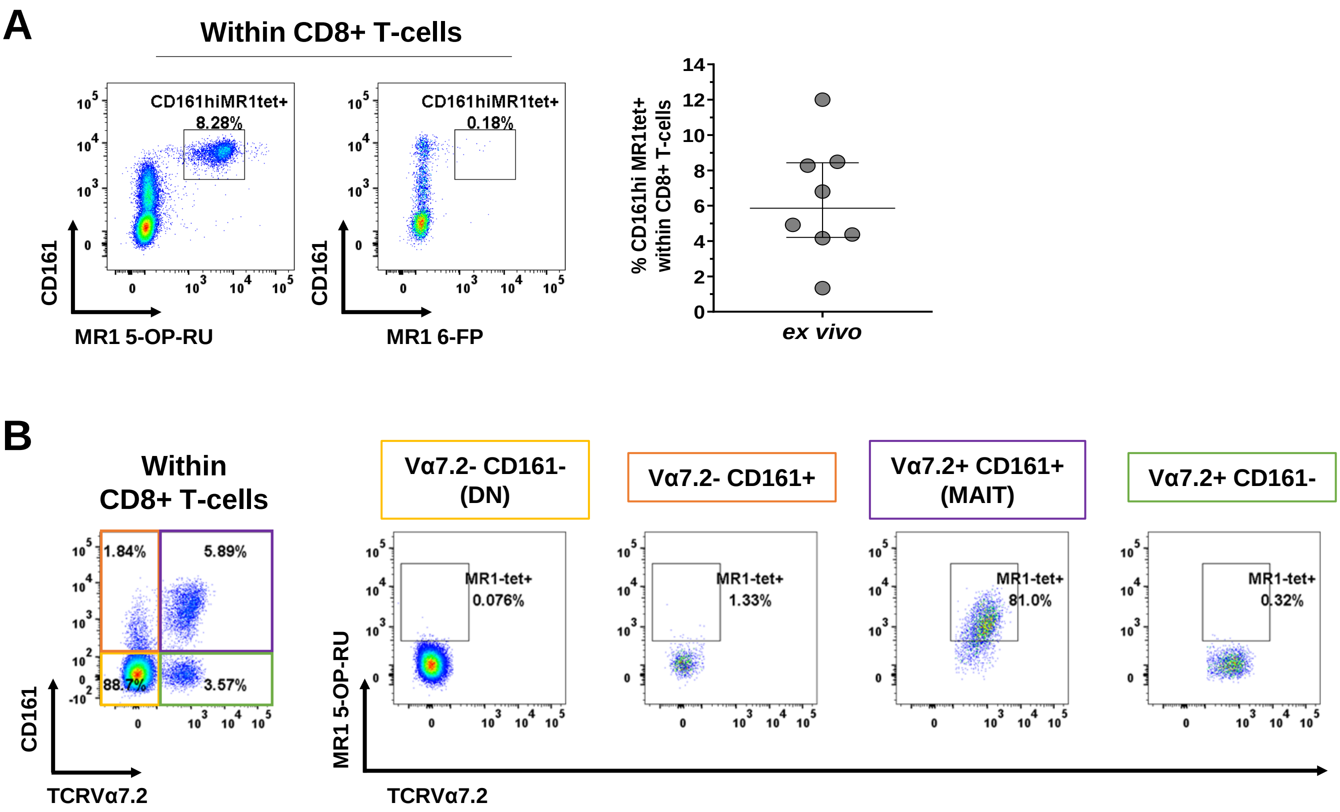

Supplementary Figure S5. Validation of Vα7.2+ CD161+ gating with MR1-5OP-RU tetramer for MAIT cell identification. **(A)** Healthy donor PBMC were stimulated *ex vivo* for 3 hours with PMA/ionomycin in the presence of GolgiStop. Representative FACS plots and cumulative data showing the *ex vivo* frequencies of CD8+ MAIT cells as identified by surface CD161 and MR1-5OP-RU tetramer staining (left panel); tetramer specificity was assessed by the negative control 6-FP loaded MR1 tetramer (right panel). Data plotted as median ± IQR (n=8). **(B)** PBMC were cultured for 3 days with plate-bound anti-CD3 mAb and soluble anti-CD28 mAb in the presence of hrIL-1β and hrIL-23. On day 3, cells were re-stimulated for 3 hours with PMA/ionomycin in the presence of GolgiStop. CD8+ MAIT cells were identified by combined surface CD161, TCRVα7.2 and MR1-5-OP-RU tetramer staining. Live CD3+CD8+ T-cells were first gated on CD161 vs TCRVα7.2 (quadrant gate) then the proportion of MR1-5OP-RU positive cells gated within each quadrant. TCRVα7.2+ CD161+ (purple) gating combination displayed an 80-90% accuracy for identification of MR1-5OP-RU+ MAIT cells. Very few MR1 tetramer binding MAIT cells were identified within TCRVα7.2+ CD161- cells (green) or TCRVα7.2- cells (yellow and orange). Staining plots are representative of a single donor.

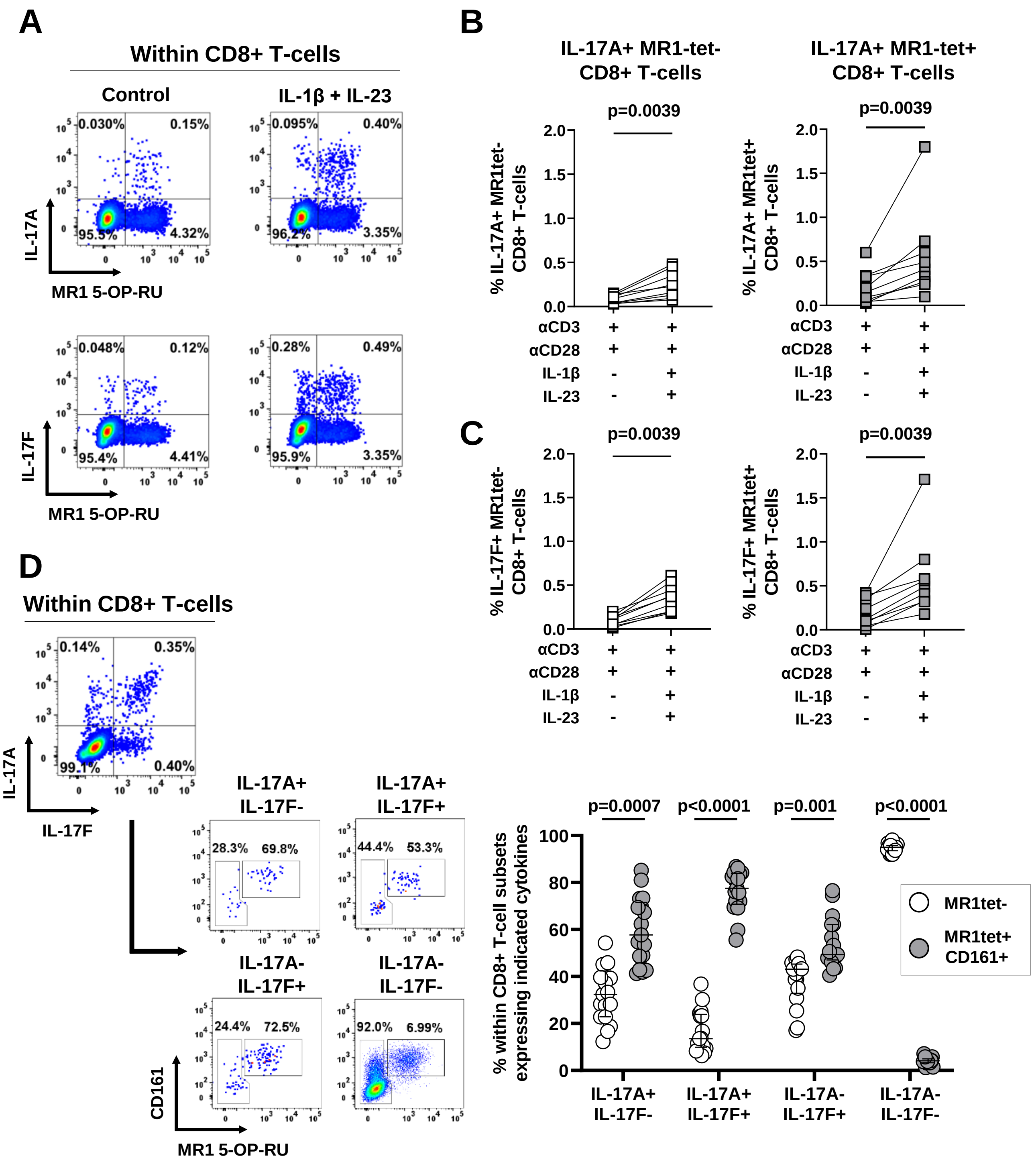

Supplementary Figure S6. Both IL-17-expressing CD8+ conventional and unconventional MAIT cells are induced within type-17 polarising in vitro cultures. Healthy donor PBMC were cultured for 3 days with plate-bound anti-CD3, soluble anti-CD28 mAbs in the absence or presence of IL-1β and IL-23 followed by 3 hours with PMA/ionomycin in the presence of GolgiStop. Representative staining plots **(A)** and cumulative data **(B, C)** show frequencies of IL-17A+ **(A, B)** or IL-17F+ **(A, C)** cells within MR1 5-OP-RU tetramer-negative (white squares) and tetramer-positive (grey squares) CD8+ T-cells (n=5). **(D)** Representative dot plots showing frequencies of IL-17A+IL-17F-, IL-17A+IL-17F+, IL-17A-IL-17F+ and IL-17A-IL17F- cells gated within total CD8+ T-cells after culture in the presence of anti-CD3/CD28 stimulation with IL-1β and IL-23, and the subsequent proportions of MAIT cells (identified by MR1 5-OP-RU tetramer and CD161 positive cells) that comprise each IL-17 cytokine expressing population. Cumulative data (n=17) are plotted as median ± IQR and statistical analysis performed using Wilcoxon matched-pairs signed rank test.

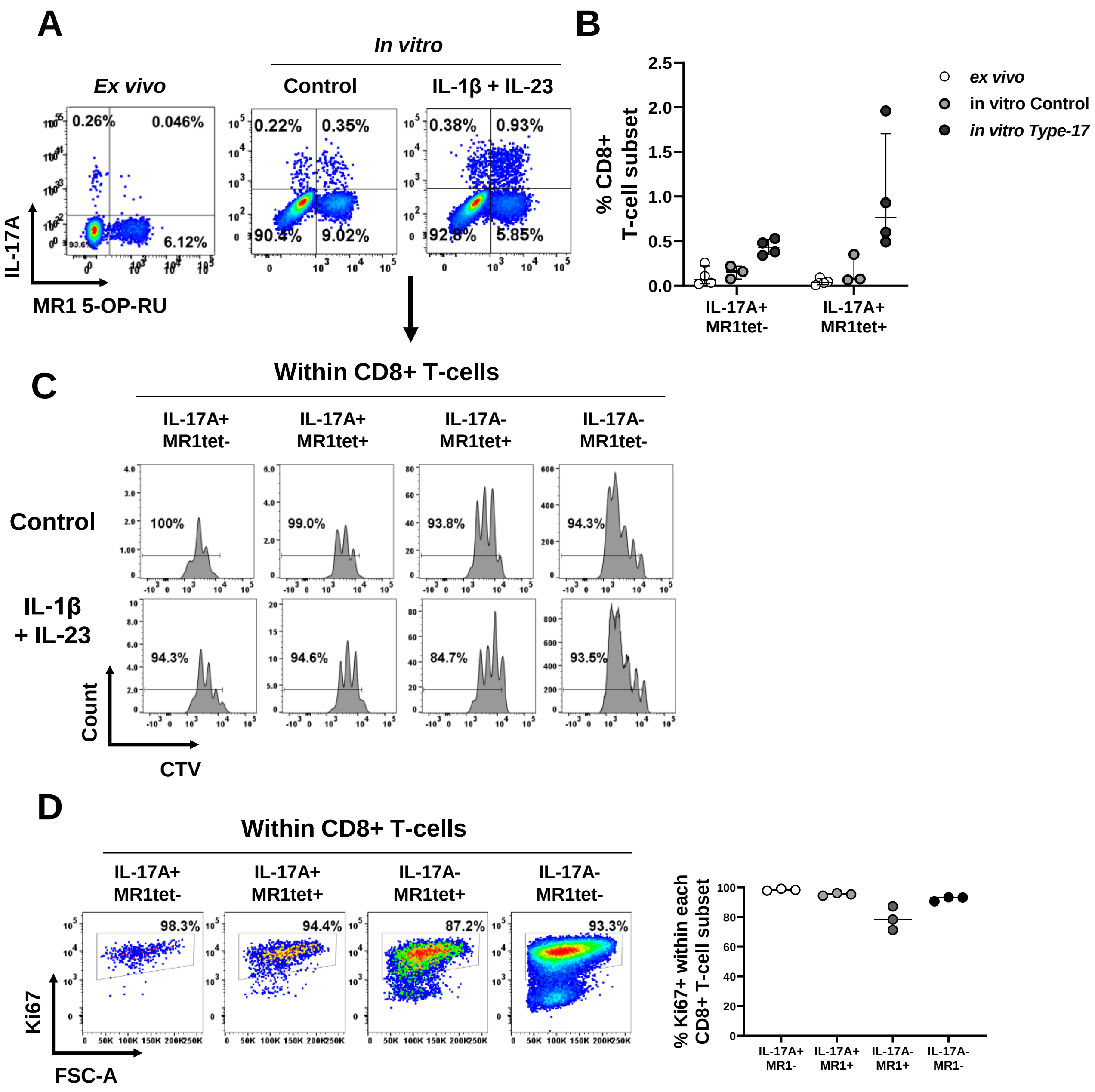

Supplementary Figure S7. *In vitro*-generated MR1 tetramer- and MR1 tetramer+ IL-17+ CD8+ T cells display comparable proliferative potential. Healthy donor PBMC were either stimulated *ex vivo* for 3 hours with PMA/ionomycin in the presence of GolgiStop or cultured for proliferative assessment. Cells were labelled with CellTrace™ Violet (CTV) then cultured for 3 days with plate-bound anti-CD3 mAb and soluble anti-CD28 mAb in the absence (control) or presence of hrIL-1β and hrIL-23. On day 3, cells were re-stimulated for 3 hours with PMA/ionomycin in the presence of GolgiStop and assessed by flow cytometry. **(A)** Representative stainings and **(B)** cumulative frequencies of IL-17A+ MR1 tetramer- and IL-17A+ MR1 tetramer+ cells identified within total live CD8+ T-cells ex vivo or after in vitro culture. **(C)** Histograms depict CTV staining and percentage of proliferating cells after control (upper panel) or type-17 polarising culture (lower panel) in the four indicated populations identified by gating IL-17A vs MR1 tetramer expression within total live CD8+ T-cells (n=1). **(D)** Representative FACS plots and cumulative data (n=3) show intracellular Ki67 staining in each denoted subset within CD8+ T-cells. Data are plotted as median ± IQR.

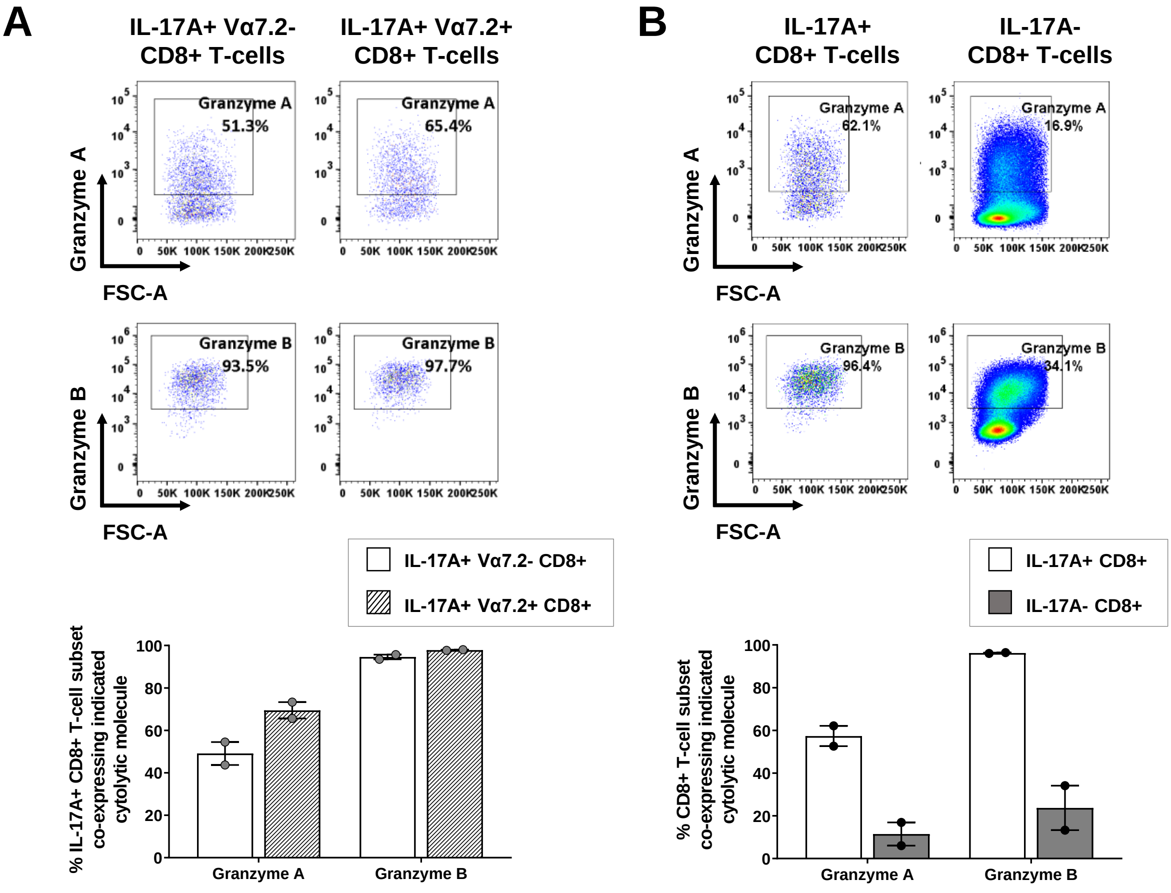

Supplementary Figure S8. Cytotoxic potential of *in vitro*-induced IL-17A+ Vα7.2- and IL-17A+ Vα7.2+ CD8+ T-cell subsets. Bulk CD8+ T-cells from healthy donor PBMC were cultured for 3 days with plate-bound anti-CD3 mAb and soluble anti-CD28 mAb in the presence of hrIL-1β and hrIL-23. On day 3, cells were re-stimulated for 3 hours with PMA/ionomycin in the presence of GolgiStop for identification of IL-17A+ CD8+ T-cell subsets and intracellular cytolytic marker expression. **(A, B)** Representative flow cytometric stainings and cumulative data (n=2) showing frequencies of IL-17A+ Vα7.2- (white bars) and IL-17A+ Vα7.2+ (hashed bars) CD8+ T-cell subsets **(A)** or total IL-17A+ CD8+ (white bars) and IL-17A- CD8+ T-cells (grey bars) **(B)** expressing granzyme A or granzyme B.
